# Dental Neuralgia

**Published:** 1879-03

**Authors:** G. V. Black

**Affiliations:** JACKSONVILLE.


AMERICAN JOURNAL

OF

DENTAL SCIENCE.

Vol. XII. THIRD SERIES—MARCH, 1879. No. 11.

### ARTICLE I.

### *Dental Neuralgia.*—Continued.

BY G. V. BLACK, JACKSONVILLE.

*Course of the Affection.*—How certain conditions predispose to neuralgia is unknown. It is equally unknown as to how exciting causes act to develop the effection. Yet inquiry as to the observed cause of observable phenomena will not be wholly unprofitable.

It is not common for the pain to be developed suddenly, as may be the case with pain from an injury. An uncertain period of time is required for its development, during which slight pains may be felt in the part about to be attacked, which gradually increase in severity. A period of preparation or development seems to be an essential feature of the affection. This is true, no matter what the exciting cause may be. A brier lodged in the flesh with its point in a nerve branch will produce a pain of a normal kind immediately upon its lodgment, but no sudden neuralgia. A neuralgia may be developed within a few days or a few weeks from the continued presence of the brier point. This is something essentially different from the normal pain accruing immediately upon its lodgment or the inflammation its presence may have excited.

We have, therefore, a development of conditions in the sensory nerves themselves, that induces and is necessary to the production of the pain in question. As before remarked, we may call this by this name or that, without arriving at any better understanding of the subject. It seems certain, however, that some nerve change is induced that gives rise to abnormal nerve function, the essential feature of which is pain; we may, therefore, designate this as the neuralgic condition of the nerves. This condition may be developed in and effect only a single branch of a sensory nerve, or it may at once be developed in all the branches of a common nerve trunk, or in a given region, or simultaneously in a single branch, or two or more branches, and then pass successively to other branches of a common nerve trunk, and then, as if by radiation from nerve center eccentrically, make its appearance in other nerve trunks in the neighborhood, and occasionally in distant regions of the body. In many instances there would seem to be little room for doubt that the spread of the affection is by propagation of the neuralgic condition along the branches and trunks of the nerves in a direction from periphery to center, and the reverse. As a rule the affection does not present a continuous progression, but after making more or less advance, becomes stationary, frequently only extending to certain trunks and its branches, or the pain may be confined to the trunk or certain portions of the trunk of the nerve. In that case the pain does not appear superficially but darts along, or is stationary, in the course of the affected portion of the nerve, as is often the case in sciatica.

The duration of the affection is exceedingly indefinite. It may continue for a lifetime, in spite of the most skillful treatment, or it may disappear within a few days. Generally, if a direct exciting cause is found and removed, the affection disappears in a very short time, if not immediately.

*Dental Neuralgia.*—Under the term *Dental Neuralgia*, we include those cases of neuralgia the direct exciting causes of which are to be found in affections of the teeth. There is no longer any doubt that neuralgia of the fifth pair frequently has its origin in diseased conditions of the teeth as the direct, exciting cause. It therefore only remains for us to point out those conditions under which this result is most likely to occur, and if possible to indicate those forms of the affection thus developed.

Of course any exciting cause of neuralgia is most liable to develope the affection in those constitutionally predisposed to it, or who have become temporarily so from any cause, as anæmia, miasma, over-work, etc. This being understood, we may proceed to discuss those conditions of the teeth tending to its development.

We should say in the beginning that neuralgia must not be confounded with those cases of toothache in which sympathetic pain is projected to more or less distance from the seat of lesion, thus causing confusion in the patient's mind as to the particular tooth which causes the trouble. These we regard as cases of confusion of the sensorium in referring the pain accurately to its proper location, so that pain in a lower molar is referred to an upper, or the reverse. Again, painin a molar may be referred to several of the adjoining teeth, as if neighboring nerve filaments had come to the aid of, and had taken upon themselves a portion of the burden of their fellow who was struggling under a load too heavy to be borne. In these cases the pain, although it may radiate to different parts of the face, is referred to the teeth as the part subjected to injury, and does not, necessarily, involve a neuralgic affection of the nerves, and usually differs essentially in its character from that affection.

At the same time we must not forget that neuralgia from causes entirely occult referred to the distribution of the fifth pair, may be felt especially in the teeth, while these organs may, in themselves, be in a perfectly healthy condition. The extraction of teeth, under such circumstances, would afford no relief, farther than temporary abatement of pain from the shock of the operation.

Miss T., a teacher, applied to me February, 1872, on account of neuralgia, of which she said the central point of pain was the right superior molar. In tracing the course of the radiation of the pain, she traced the second division of the fifth pair, and that only. The molar tooth, and a point below the orbit—deep seated—were the especial points of pain. We noticed that in pointing out the course and seat of pain, she avoided touching the face. On brushing our fingers lightly across the face, a paroxysm of pain at once occurred.

Here were two points that very clearly contra-indicated a dental origin of the affection: 1st, It had been in progress for six weeks, and only the second division of the fifth pair was involved. In every case of demonstrated dental neuralgia we have seen, the second and third divisions have been at once or very quickly involved. 2nd, The act of conduction of sensation from the surface induced a paroxysm, which we have never seen in dental neuralgia.

The tooth in question had a very large gold filling in the grinding and mesial surfaces, which he had placed there four years before, after removing the pulp. It showed no sign, whatever, of pericemental disease. The patient was evidently suffering from overwork and anæmia. We advised rest and a tonic course of treatment, both of which were practically disregarded. The patient was anxious to have the tooth out, which we refused, fully believing it would be of no benefit. The case grew rapidly worse, and extended over a considerable part of the body, becoming bilateral. She was forced to leave her duties, and resolved to have the tooth out. She applied to another operator and had it removed. The case continued to grow worse. After exhausting her physician, she was advised, as she afterwards expressed it to me, " to go to the mountains and go wild in the woods," which she said she accomplished to the letter in a double sense; wild with pain and wild in the mountains. After two years of suffering, she slowly improved and was restored to her former health.

Dental Neuralgia we believe to arise from, and to be symptomatic of certain lesions of the dental pulp. So far as we have personally observed, it makes its appearance first in the fifth pair, and uniformly affects some portion of both the second and third divisions. From these it may extend to the first division, to the nerves of the neck and chest, and to the brachial plexus and upper arm. It seems never to appear superficially on the skin, but is always deep-seated, seemingly confined to the nerve trunks and principal branches—otherwise it does not differ especially from neuralgia of the fifth pair from other causes.

The affection is never developed directly from acute inflammation of the tooth-pulp. In this condition pain may be radiated to neighboring parts, the same as sympathetic pains may arise in the neighborhood of other inflammations. How much this may have to do with the final development of neuralgia in those predisposed to it, is uncertain. Our own observation is, that very few sharp, active inflammations of the tooth-pulp degenerate into neuralgia, yet we have seen examples where this seems to have been the case. In all such, the inflammation has first become chronic and the local pain mostly or entirely abated before the occurrence of prominent neuralgic symptoms. As a rule, hyperæsthesia of the pulp precedes the occurrence of the affection, and it has generally been noticed by the patient that a certain tooth has been sensitive to thermal changes sometime previous to the attack, and perhaps for a time after the development of the affection. This usually gives way, gradually, to an anæsthetic condition of the pulp. In very many cases we have examined, however, no such previous or accompanying hyperæsthesia could be discovered, the patient being positive that there had been no abnormal sensitiveness of the teeth. In such cases it is rare to find the offending tooth in the front, or an exposed part of the mouth. A common situation in such instances in which to find the exciting cause is in a second molar or wisdom tooth, with a buccal decay about the margins of the gums, with a fungous growth of gum growing into and filling the cavity of decay, very effectually protecting the exposed pulp from external influences. From these circumstances, we are convinced that hyperæsthesia of the pulp is always a forerunner of neuralgia, but it is not necessarily great, and may often escape the notice of the patient, provided the cavity be occluded and in the posterior of the mouth. By far the larger portion of dental neuralgia arises from exposure of the pulp in protected situations, where it remains uninjured by foreign bodies, and vitiated secretions are retained in contact with it. It seems likely that this circumstance is in part necessary to the changes which must occur in the pulp as a step in the initiation of that nerve change which must begin at this point and proceed thence along the branches and trunks of the nerves, in order to the development of the affection. Such teeth are not usually sore to the touch, nor to rapping with an instrument. This may, in some instances, excite a paroxysm of neuralgia, but our observation induces us to believe this is rare. Therefore, we consider this test, so much relied on by physicians, and so often reported in medical writings, as wholly unreliable in proving or disproving the dental origin of the affection.

After a few days' existence of neuralgia, if such a pulp be touched with an instrument it will be found sensitive, perhaps abnormally so, and a paroxysm of pain will generally follow the experiment. But if the neuralgia has been in existence for several weeks and has been severe, the pulp may be found in a state of complete anæsthesia, yet a neuralgic paroxysm is likely to follow within a few minutes after a puncture. How long a pulp may remain in this condition and yet retain its vitality, is unknown, and is probably very indefinite. We have seen several cases in which it had existed three months, and one in which we have reason to believe it had existed more than six months, during all of which time the paroxysm had been frequent and very severe. We will relate the case:

April 20th, 1871, Mrs. L. N., aged about thirty, married, applied for removal of second right superior bicuspid, which was very much decayed, being directed by her physician to have it out, on account of neuralgia. She gave the following history: About six months ago she had some difficulty with her teeth, and as the molars and first bicuspid on this side were badly decayed, she had them removed. The second bicuspid was also much decayed, but she intended to have it filled, but had neglected it. She had no more trouble with her teeth, but continued to have occasional paroxysms of neuralgia, in both upper and lower jaw, which increased in frequency and severity, and gradually extended to temple and brow, and to the orbit; and also had severe ear ache. Within about six weeks the pain extended down the side of the neck, and finally to the chest, afterwards to the upper arm. It had been so severe in this position that she could not use her arm well. She had no superficial pain at any time; no prickling or loss of sensation, in the skin. The patient was very pale; decidedly anæmic, and seemed in a very weak condition of body. Stated that her health had been good before, though she was not robust.

Examination of the bicuspid tooth showed a large decay in mesial surface, pulp dead, peridental membrane healthy. A decay was discovered in the cuspid, mesial surface, with opening into pulp, which seemed insensible, but bled on being touched with the excavator.

We proposed the removal of this pulp, to which patient objected, saying she had been sent to have the bicuspid removed. This we refused, and referred her back to her physician, with statement of the result of our examination. 21st. Patient returned. While opening the pulp chamber for removal of pulp, a very severe paroxysm of pain occurred throughout the affected parts. Arsenic was applied.

23d. Reported very severe suffering for several hours after application. 24th. Better, but objected to having pulp removed, feared another paroxysm. As there was no signs of soreness, it was deferred until next day. 25th. Removed pulp without exciting pain. No paroxysm had occurred for twenty-four hours. Patient rapidly recovered her usual health.

In another case: a woman aged about twenty-five, unmarried, a seamstress, before in apparent good health, came to me with neuralgia that had proved intractible for three months. The history of the case showed the affection to have made its appearance in the third division of the fifth pair, pain quickly involving the second, and more gradually spreading to other nerves, until the branches of the fifth, the side of the neck and chest were involved, but not the arm. There was in this case considerable mammary pain, which, however, had been the last locality in which pain had been developed.

The cause of the difficulty was found in a lower molar, the pulp of which I had capped with oxychloride of zinc and filled some weeks before the occurrence of the neuralgia. The pulp was at once removed by the broach. In itself it did not seem sensitive, but a severe paroxysm of neuralgia followed. Within a week the paroxysm disappeared, but the anæmic headache, a severe pain in the top of the head, persisted.

We saw the patient no more, but learned that malposition of the womb occurred during this anæmic state, and chronic metritis was developed, from which the patient made a tedious and imperfect recovery.

These cases will usually, if the neuralgic predisposition be not very decided, be relieved by the destruction of the irritated tooth-pulp, or removal of the tooth, without other treatment. Spontaneous death of the pulp is attended with a like result.

The cases we have recited have been the worst forms we have met with. Cases of such duration, we are persuaded, are not very common, nor is there any necessity for their duration, if the cause be recognized. Most of the cases we have met with, have been of only short duration. That some of these would have been as bad as those related, if the cause had not been recognized, we have every reason to believe.

*Pathological Condition of Pulp.*—In these cases the condition of the pulp, as before remarked, is never that of active inflammation. In the cases where we have minutely examined the pulp we should explain it, first, as a destructive hyperæmia; there is an abnormal amount of blood without, as a rule, much hyperæsthesia, and second, what is of importance as showing the tendency of this diseased action, the dentinal fibrils have lost their sensibility, as shown by the entire absence of sensibility in cutting into the dentine. We have examined no well-marked case of neuralgia from the tooth-pulp where this was not the case. Third, there is often found a marked disposition to destructive ulceration of the pulp. The exposed surface in this case is usually covered with pus; this condition, as is the case with most others recited, may often be met with where there is no neuralgia. Fourth, minute abscesses or pockets of pus are frequently found within the substance of the pulp. We have frequently discharged a minute drop of pure pus from a pulp and in a few instances have repeated this several times at intervals of a few days. Fifth, the pulp may be a little reddened, a natural color, or even ashy in appearance, not giving much evidence of disease by ordinary examination. When we have been able to remove and make microscopical examination at once, the tissues have been literally packed with wandering cells or leucocites. The number of these cells that may be present in the tissue without appearing distinctly as pus has greatly surprised me, and leads to the inference that this is essentially the condition of the pulp in all these cases of which the phases before described are modifications. How this may be we are not prepared to state, as this is a matter of recent observation. In two instances we have been able for a few minutes to see distinctly the motions of these cells as they crept, amœba like, through the tissues. This is a matter of great interest to both the Pathologist and the Microscopist, and we hope others will make observations. It seems likely that there is a tissue condition in which it becomes crowded with wandering cells to the extent, perhaps, of considerable swelling, without or almost without, the other phenomena of inflammation.

*Pericementum.*—Our observation leads us to believe that neuralgia is never induced by pulpless teeth or pulpless roots. We are perfectly aware that many reports may be found in both dental and medical literature, that would seem averse to this opinion. But in closely scanning these reports we do not find that the conditions of such roots have been reported with such accuracy as would be necessary to establish that the neuralgia was a result of pericemental irritation

Most of them are absolutely silent as to any definite examination that would determine whether or not a living pulp was present, and are, therefore, worthless so far as this point is concerned. We have never yet seen a case of neuralgia that could clearly be referred to, or was relieved by the removal of, pulpless roots or pulpless teeth. We have, however, met with a case that would doubtless have been so considered, if the examination had been less rigid. This was a first inferior molar—crown gone and an abscess at the anterior root discharging upon the gum. The broach revealed a living pulp in the posterior root, which bled freely, and the puncture of which was followed by a paroxysm of pain.

As to why peridental inflammation should fail to induce neuralgia, while such symptoms are induced by pulpal irritations, we have no theory to offer. This opinion rests entirely upon observed facts. This class of irritations begets another class of phenomena, which we may call projected sensations, or sympathetic pain in various regions, which are always co-existant with pain at the seat of disease. They are usually accompanied by disturbances of the lymphatics, and the glands are often swollen and painful, which is rarely an accompaniment of pulpalgia or neuralgia.

*Calcific Nodules.*—What the influence of calcific nodules in the pulps may be in exciting neuralgia is difficult to determine. Our own observation would be against the production of neuralgia from this cause. But that the sharp corners thus formed may serve to perpetuate irritations aroused by other causes scarcely admits of a doubt.

*Exostosis.*—Exostosis of the roots of the teeth, or more properly, hypertrophy of the cementum, is undoubtedly an occasional cause of neuralgia of the fifth pair, but we are persuaded that it is of rare occurrence; we have never met with but one case that could clearly be referred to this cause. This we had under observation more or less closely for more than a year. The pain was felt mostly in the lower jaw, but the ear, a location deep-seated under the eye, and the malar prominence were often referred to as painful. Before the case came under our observation, most of the teeth on that side in the lower jaw had been removed in the vain attempt to cure the trouble, but without effect. We finally suspected a superior molar that had protruded from loss of its antagonist; upon removing it the roots were found largely exostosed—tooth otherwise healthy. Complete and permanent cessation of the neuralgia, within a week, which had been of five years' duration justifies the opinion that the exostosis was the cause.

Quite a number of reports of neuralgia from this cause are found in our literature. We know of no distinctive feature that can serve us in arriving at a diagnosis.

*Alveolar Abscess.*—Neuralgia from the burrowing of an alveolar abscess into the inferior dental foramen is an occasional occurrence. This is generally met with in the chronic form of abscess and, in the two cases coming under observation, the region supplied by the nerves escaping from the mental foramen and the anterior teeth have been the seat of pain. In such cases the presence of a tumor or a fistulous opening on the face, or more generally beneath the maxilla, will point unerringly to the source of the mischief. One of our cases was rather interesting. Mr. Geo. A. applied to me with neuralgia of the chin and lip on the right side, which had recently appeared also in the anterior teeth. Patient stated that it had troubled him more or less for eight or nine years, but he was frequently free from it for several months at a time. The paroxysms were sometimes severe, but on the whole it would not be considered a severe case. Examination of the mouth revealed a row of perfect teeth upon the lower jaw of this side, except that the first molar was gone. The gums, at the point where it was removed, appeared healthy. A partially cicatrized fistula was found under the maxilla, about midway between the mental foramen and the angle. A probe started into this was carried forward and upward directly through the bone until it almost reached the gum where the tooth had been removed. The gums were dissected away and the bone cut into with a chisel—it was but a shell—disclosing a cavity in which was found a root of the missing molar, and some fragments of necrosed bone.

We should have stated that the missing molar had been removed eight years before for the cure of this fistula which, having failed, other means also failing, the condition had become to be considered by the patient as incurable.

*Crowding of Teeth*, and impacted teeth, may possibly cause neuralgia by causing compression of the dental nerves in their bony canals. We know of no means of recognizing a cause of this nature. Examinations of each inidvidual case must be conducted by the judgment of the operator and the relations of the teeth studied. The probable position of impacted teeth made out as nearly as practicable, in order that their influence may be estimated. We are unable to give any estimate as to the frequency that these have been demonstrated to have been the cause of neuralgia.

The cases that have already found their way into our literature have generally been too indefinitely reported to serve as a basis for conclusions. We have never seen a case that we could clearly attribute to impacted teeth, and but few where crowding of the teeth seemed to act as a cause, and these were not very clearly made out.—*Transactions of Ill. Dental Society*

---

